# Seed production of wild soybean (*Glycine soja* Sieb. *et* Zucc.) under favorable, ruderal, and natural growing conditions

**DOI:** 10.1371/journal.pone.0274668

**Published:** 2022-09-29

**Authors:** Aki Mizuguti, Daisuke Aoki, Kei Takamoto, Aya Arii, Hidetoshi Goto, Shuichi Nakai, Michael J. Horak, Keguo Huang, Duška Stojšin

**Affiliations:** 1 Faculty of biotechnology, Fukui Prefecture University, Futaomote, Awara City, Fukui, Japan; 2 Previously Faculty of biotechnology, Fukui Prefecture University, Kenjojima, Matsuoka, Eiheiji cho, Fukui, Japan; 3 Bayer Crop Science K. K., Marunouchi, Chiyoda-ku, Tokyo, Japan; 4 Previously Bayer Crop Science K. K., Marunouchi, Chiyoda-ku, Japan; 5 Previously Monsanto Company, St. Louis, MO, United States of America; 6 Bayer Crop Science, St. Louis, MO, United States of America; National Agricultural Research Centre, PAKISTAN

## Abstract

Field trials were conducted in Japan under different growing conditions to better understand seed production of wild soybean (*Glycine soja* Seib. *et* Zucc.). The objectives of these trials were to evaluate yield and yield components of wild soybean: (1) across 11 diverse populations grown under favorable conditions to assess seed production potential, (2) under different planting densities (112, 208, 416, and 832 plants/m^2^) to assess intra-specific competition, and (3) across growing conditions (favorable, ruderal, and natural) to assess the impacts of environmental stress and inter-specific competition. Significant differences in yield and seed number observed among the evaluated wild soybean populations were predominantly due to environmental effects and genetic by environmental interaction. Seed production was impacted by both intra- and inter-specific competition. Wild soybean grown without plant competition had 51-fold and 247-fold higher yield compared to when grown in ruderal and natural environments, respectively. Under favorable growing conditions, wild soybean had substantial yield potential due to the ability to produce a high number of seeds. In nature, yield potential is severely limited because of plant competition and other environmental stressors. The results of this research are useful to inform environmental risk assessment when considering the potential impact of soybean biotechnology traits that increase or protect yield. If such traits were to be inadvertently transferred from imported soybean into wild soybean, this research indicates that the effects would likely have little overall impact on wild soybean seed production.

## Introduction

Wild soybean (*Glycine soja* Seib. *et* Zucc.) is the closest relative of the cultivated soybean (*Glycine max* L. Merr.), which was domesticated in eastern Asia [1–3]. Both species are highly self-pollinated with low outcrossing rates resulting in a very low frequency of inter-specific hybridization in nature [4–6]. However, considering that outcrossing can occur, albeit at very low levels, the evaluation of the potential effect of soybean biotechnology traits on wild soybean is informative to an environmental risk assessment (ERA) for regions in Asia where wild soybean grows naturally.

In the USA, annual yield of soybean has increased more rapidly since transgenic varieties were released than during the prior period when farmers grew non-transgenic soybean only [7]. To assess the potential impact of a soybean biotechnology trait that increases yield or protects against yield loss in soybean, it is important to understand the factors that influence seed production of wild soybean.

Wild soybean typically grows in natural habitats (with established plant communities) or in ruderal areas (along rivers, in and around cultivated fields, or where soil is disturbed by floods or human activities) [8–10]. In these habitats, wild soybean is exposed to environmental stressors like plant competition, abiotic factors, herbivory, and diseases, each impacting wild soybean growth and development [3,11] and consequently decreasing seed production.

Intra-specific competition under high plant density has been shown to result in plant suppression or death through the process of self-thinning [12]. For cultivated soybean, self-thinning was observed for densities over 115 plants per m^2^ [12] and was associated with reduced plant survival [13]. Increased plant density also gave rise to fewer branches [14,15], higher rate of leaf loss [15], and ultimately reduced yield [13,15]. The decrease in yield was due to fewer pods or seeds per plant [13–16], whereas the seed size was generally unchanged [14,15].

Inter-specific competition, as evidenced by strong competitive pressure from other plant species, can cause a substantial decrease in the number of wild soybean plants, resulting in a reduced survival rate ranging from 0 to 63% [3,11]. In addition to plant competitive pressure, abiotic factors such as flooding, have also been shown to decrease wild soybean survival in nature to 12–39% [11]. Other environmental factors affecting natural habitats, such as herbivory, can result in foliar damage ranging from 3.6 to 30.6% [17]. Even though wild soybean plants compensate for the reduction of leaf tissue, excessive defoliation may result in decreased seed number [17].

Evaluations under favorable, nearly stress-free growing conditions are informative as plants can express their seed production potential [18]. However, information regarding the seed production potential of wild soybean is limited. Consequently, the extent of the reduction in seed production under ruderal and natural growing conditions has not been fully explored.

The objectives of this study were to evaluate yield and yield components of wild soybean (1) across populations grown under favorable conditions to assess their seed production potential, (2) across different planting densities under ruderal and natural conditions to assess the effects of intra-specific competition, and (3) across different growing conditions (favorable, ruderal, and natural) to assess the effects of environmental stressors that may occur in nature, including inter-specific competition. The results of this research will provide insight into the relative importance of environmental stressors on the seed production of wild soybeans under natural conditions. Furthermore, the results will be useful for ERA of soybean biotechnology traits that increase or protect yield if inadvertently transferred from imported soybean into naturally growing wild soybean.

## Materials and methods

### Trials under favorable growing conditions

Eleven wild soybean populations originating from four Japanese prefectures (Aomori, Ibaraki, Hiroshima, and Saga) were evaluated in this study (Table 1). The seed sources for the Ibaraki and Saga populations came directly from samples collected from natural habitats. Plants sharing similar phenotype, growing together and away from other wild soybean plants were considered to constitute a population. On average, 2,567 seeds were collected per population. The starting seed of Aomori 1 and Hiroshima populations originated from the genebank of the National Institute of Agrobiological Sciences (NIAS), whereas the Aomori 2 population was selected from Aomori 1 and provided by Dr. Saji (National Institute for Environmental Studies, Tsukuba, Ibaraki, Japan). Even though Aomori 1 and Aomori 2 originated from the same population, Aomori 2 is a subset of Aomori 1 and as such represents a more genetically uniform population.

**Table 1:** Details regarding wild soybean populations.

Population	Collection site coordinates		Seed source ^a^
	Latitude (N)	Longitude (E)	
**Aomori 1**	40˚ 1’ 6”	141˚ 2’ 5”	JP 36034
**Aomori 2**	40˚ 1’ 6”	141˚ 2’ 5”	JP 36034 selection
**Ibaraki 1**	36˚ 1’ 7”	140˚ 4’ 5”	Natural population
**Ibaraki 2**	36˚ 1’ 7”	140˚ 4’ 5”	Natural population
**Ibaraki 3**	36˚ 2’ 0”	140˚ 3’ 6”	Natural population
**Ibaraki 4**	36˚ 3’ 10”	140˚ 3’ 8”	Natural population
**Ibaraki 5**	36˚ 9’ 5”	140˚ 3’ 2”	Natural population
**Hiroshima**	34˚ 4’ 4”	133˚ 7’ 7”	JP 110755
**Saga 1**	33˚ 6’ 2”	130˚ 9’ 4”	Natural population
**Saga 2**	33˚ 6’ 8”	130˚ 2’ 0”	Natural population
**Saga 3**	33˚ 8’ 6”	130˚ 6’ 7”	Natural population

^a^ Aomori 1 and Hiroshima originated from NIAS accessions JP 36034 and JP 110755, respectively. Aomori 2 originated from NIAS population JP 36034 that was selected and maintained by Dr. Saji.

Field trials were conducted in two consecutive years (2012 and 2013) and at two prefectures (Ibaraki and Fukui). The four growing environments were designated as 2012IB1, 2012IB2, and 2013IB for trials conducted at Ibaraki prefecture, and 2013FU for the trial at Fukui Prefecture University (Table 2). The Aomori 2 population was tested in 2013 only, whereas the other populations were tested in both years. The wild soybean populations (10 in 2012 and 11 in 2013) were planted in plots arranged in a randomized complete block design (RCBD) with 18 replications per site. Five scarified seeds (seed coat abrasion with sandpaper) were planted in the center of the 1 m^2^ (1 m x 1 m) plot. The alleyways between replication blocks were approximately 1.5 m wide.

**Table 2:** Planting and harvest dates for trials in 2012 and 2013.

Location	2012IB ^a^		2013IB		2013FU
**Favorable growing conditions**					
Planting date	May 14, 2012	May 16, 2012		Jun 4, 2013	Jun 18, 2013
Harvest date	Oct 5–31, 2012	Oct 9 –Nov 5, 2012		Oct 4—Nov 5, 2013	Oct 9—Nov 1, 2013
**Ruderal growing conditions**					
Planting date	Jun 29, 2012		Jun 11, 2013		Jun 28, 2013
Harvest date	Oct 19, 2012		Oct 11–17, 2013		Oct 9–15, 2013
**Natural growing conditions**					
Planting date	Jun 29, 2012		Jun 11, 2013		Jun 28, 2013
Harvest date	- ^b^		Oct 11–17, 2013		No plants survived

^a^ In 2012 at Ibaraki, there were two sites (2012IB1 and 2012IB2) under favorable growing conditions.

^b^ Not harvested due to early pod shattering.

Agronomic practices typical for soybean cultivation were conducted throughout the season. Prior to planting, the study area was tilled. Root nodule bacteria (Dr. MAMETARO, Idemitsu Kosan Co., Ltd) were applied to the soil at 200 kg/ha at planting. Chemical fertilizer was also applied at planting at the following rates: 30 kg/ha of N, 100 kg/ha of P_2_O_5,_ and 100 kg/ha of K_2_O. Entries were thinned to a single plant per plot at V2—V3 stage. Rainfall during the season was adequate for wild soybean growth and development at the 2012IB1, 2012IB2, and 2013FU sites, whereas the 2013IB site was irrigated during the flowering period. Insecticides and fungicides were used as needed to control insect pests and diseases. At the 2013IB site, some damage by Hemiptera sp. was observed, however none of the plots were lost due to this pest. Weeds were controlled by hand-weeding as necessary and by using a black tarpaulin. Bird netting was used until the first trifoliate leaf stage to prevent bird damage. At 2012IB2, some feeding damage by rabbits was observed across several plots early in the season. A fence was erected around the trial site to prevent further damage by rabbits and other small animals.

To limit plot-to-plot plant competition, support stakes were placed in the middle of the plots to allow each wild soybean plant to twine. In addition, 1 m high fencing was erected on the outside of the outer plots, as well as between adjacent plots to prevent intertwining of vines from different populations in 2013. At 2013FU, several plots had plants with some intertwining of lateral branches from the adjacent plots. These branches were either untangled or cut.

Plant flowering and maturity were expressed as number of days after planting (DAP) to flowering or to maturity (when approximately 30% of pods turned black), respectively. The plants were hand-harvested from October to November (Table 2) prior to pod shattering to avoid seed loss. The harvest period extended across several weeks at each site. After harvest, individual plants were dried and threshed. Yield was measured as the total weight (g) of seeds per plant. Seed number represented the total number of seeds per plant, whereas seed size was expressed as the weight (g) of 100 seeds.

### Trials under ruderal and natural growing conditions

The wild soybean population designated Aomori 1 was also evaluated under sub-optimal growing conditions. Field experiments were conducted in 2012 and 2013 at Ibaraki and in 2013 at Fukui prefecture locations. At each site, trials were planted under two different growing conditions: ruderal (*i*.*e*., disturbed areas) and natural (*i*.*e*., areas with established plant communities). The locations were designated as 2012IB and 2013IB for trials conducted in Ibaraki prefecture and 2013FU for a trial at Fukui Prefecture University (Table 2). The trials under ruderal conditions were initiated in weed-free fields where wild soybean was planted with Japanese millet (*Echinochloa esculenta* (A. Braun) H. Scholz) in 2012 and with Italian ryegrass (*Lolium multiflorum* Lam.), perennial ryegrass (*L*. *perenne* L.), and white clover (*Trifolium repens* L.) in 2013. These species were planted uniformly and at the same rate across all the ruderal plots within each site. The fields with natural growing conditions used in this study were adjacent to ruderal trials. These were fields with a natural plant community already established. Sub-plot areas were mowed prior to trial initiation.

Under both ruderal and natural conditions, wild soybean was planted in plots arranged in RCBD with four replications per site. The size of each plot was 9 m^2^ (3 m x 3 m). Scarified wild soybean seeds were planted at equidistant spacing in a 0.06 m^2^ (25 cm x 25 cm) sub-plot in the center of each plot at four seeding densities (7, 13, 26, and 52 seeds/sub-plot), corresponding to planting densities of 112, 208, 416, and 832 seeds/m^2^, respectively. Insecticide and insect repellent were used at planting to prevent insect damage of seedlings. Bird netting was used until the first trifoliate leaf stage to prevent bird damage. At Ibaraki, a fence was placed around the trials to prevent plant damage by rabbits and other small animals. Besides these measures, the fields were not actively managed against pests to closely mimic the ruderal and natural environments.

Wild soybean characteristics evaluated in the ruderal and natural environments included plant number, leaf coverage, yield, seed number, and seed size. Plant number and leaf coverage data were collected four or five times during the season at approximately 2, 4, 8, 12, and 16 weeks after planting or until wild soybean matured. Yield, seed number, and seed size were collected at the end of the season. Plant emergence (%) was expressed as a proportion of wild soybean seeds that germinated and emerged approximately two weeks after planting. End of season survival (%) was calculated as a proportion of emerged wild soybean plants that survived and were harvested at the end of the season. Vegetation other than wild soybean were evaluated for leaf coverage only. Leaf coverage (%) (for both wild soybean and other vegetation) was estimated as a proportion of a sub-plot covered by plant canopy.

The trial grown under natural conditions at 2012IB could not be harvested because of early pod shattering, but wild soybean emergence, plant number, and leaf coverage, as well as leaf coverage for other vegetation were collected throughout the season. The trial grown under natural conditions at 2013FU site could not be harvested as no plants survived to the end of the growing season due to natural, sub-optimal environmental conditions. All other trials were harvested in October (Table 2). After harvest, seeds were dried, and yield (total weight of seeds per plot) was determined. Seed number represented a total number of seeds per plot, whereas seed size was expressed as the weight (g) of 100 seeds. Yield and seed number were also expressed on a per plant basis.

### Statistical analyses

For 11 wild soybean populations grown under favorable conditions, the SAS PROC MIXED program was used to conduct an analysis of variance (ANOVA) for the measured characteristics [19]. The ANOVA model considered the wild soybean population as a fixed effect, while environment, genotype (population) by environment interaction (G x E), and replication within environment as random effects. All comparisons among wild soybean populations were tested at the 0.05 significance level. The SAS PROC MEANS program was used to calculate the means and standard errors (SE) for each wild soybean population across environments [19].

Variance component analysis was used to assess the variation associated with the measured characteristics. The SAS PROC MIXED was used to estimate the variance parameters for genetic, environmental, and G x E interaction. The variance estimate of each component was expressed as a proportion of the total variance.

For the Aomori 1 population grown under ruderal and natural conditions, the SAS PROC MIXED program was used to conduct ANOVA analysis considering growing condition, plant density, and observation time for the measured characteristics [19]. The heterogeneous variance among growing conditions was used for proportion and weight variables to account for heteroskedastic nature of the data. The cubic root variance stabilizing transformation was used for plant number. All comparisons among plant density groups were tested at the 0.05 significance level. SAS PROC MEANS program was used to calculate the means and SE for each plant density group [19].

For the Aomori 1 population grown under favorable, ruderal, and natural conditions, the SAS PROC MIXED program was used to conduct ANOVA analysis for yield, seed number, and seed size [19]. All comparisons among growth conditions were tested at the 0.05 significance level. The SAS PROC MEANS program was used to calculate the means and SE for each evaluated characteristic [19].

## Results

### Comparison across populations under favorable conditions

**Fig 1:**
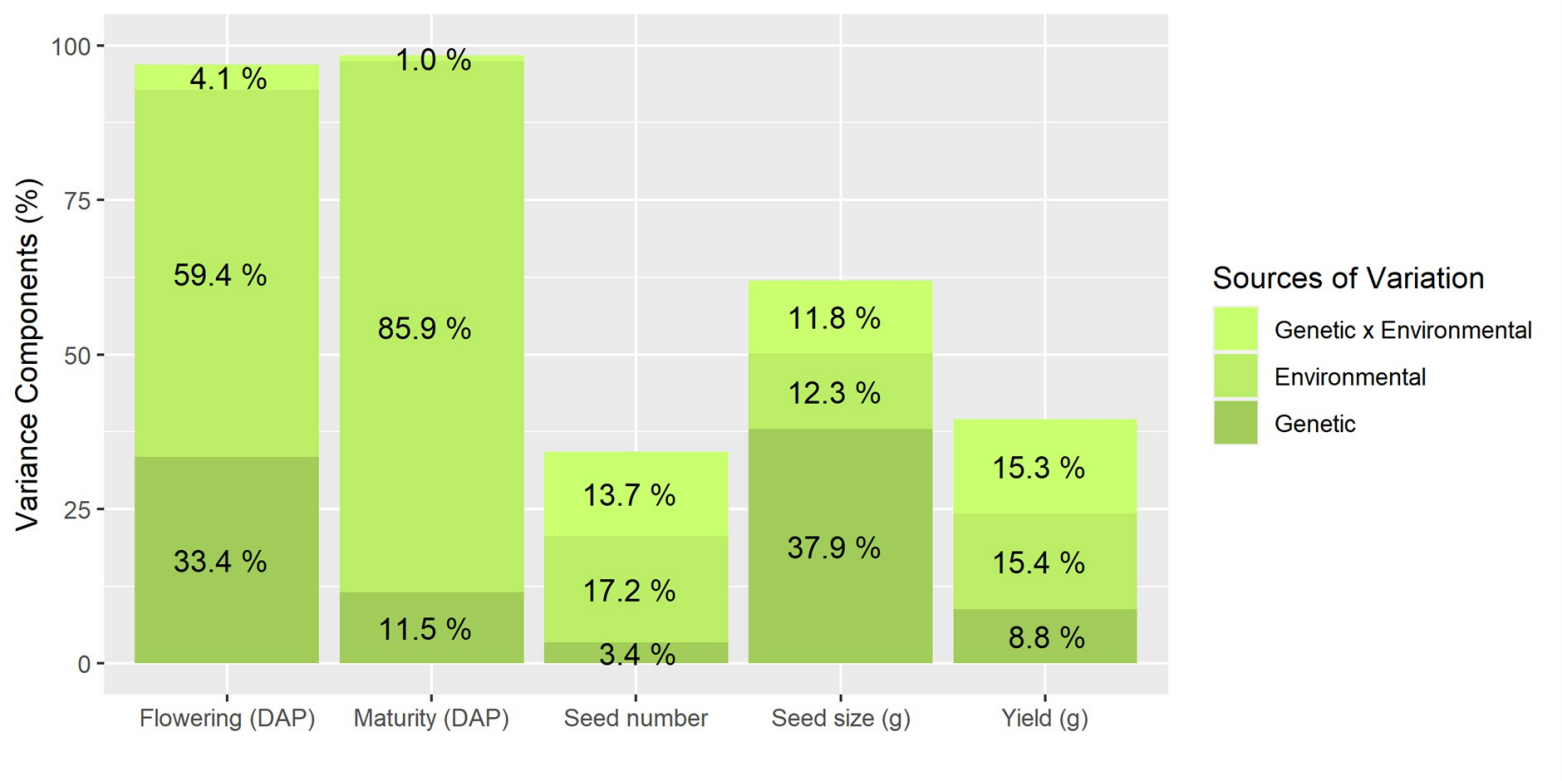
Estimates of genetic, environmental, and genetic-by-environmental (G x E) variance components (%) for wild soybean flowering, maturity, seed number, seed size, and yield.

Large variation and significant differences in flowering and maturity were observed across populations. Flowering ranged from 70.0 to 104.3 DAP, whereas maturity ranged from 120.1 to 155.6 DAP (Table 3). Variance component analysis indicated contribution of environmental (59.4% and 85.9%) and genetic factors (33.4% and 11.5%) for flowering and maturity, respectively (Fig 1).

**Table 3:** Wild soybean values (mean±SE) for flowering, maturity, yield per plant, seed number per plant, and seed size under favorable growing conditions.

Population	Flowering (DAP) ^a^	Maturity (DAP) ^a^	Yield (g)	Seed number	Seed size (g) ^b^
**Aomori 1**	74.2±1.3 d	133.9±1.9 e	271.8±15.7 ab	7,626±404.2 bc	3.6±0.1 a
**Aomori 2**	70.0±1.4 d	120.1±1.2 e	188.2±12.2 bcd	7,217±510.4 bc	2.7±0.1 cde
**Ibaraki 1**	94.7±1.2 b	148.8±2.0 bc	263.6±10.0 ab	8,970±285.1 ab	2.9±0.1 bcd
**Ibaraki 2**	96.0±1.1 b	150.5±2.1 b	275.3±9.7 a	8,845±264.9 ab	3.1±0.1 bc
**Ibaraki 3**	97.9±1.2 b	155.3±1.9 a	240.7±10.3 abc	8,292±294.4 abc	2.8±0.1 cde
**Ibaraki 4**	94.5±1.3 b	149.1±2.2 bc	233.2±9.4 abcd	9,087±373.9 ab	2.6±0.1 e
**Ibaraki 5**	88.3±1.0 c	143.5±2.0 d	179.8±6.9 d	7,162±276.7 c	2.5±0.0 e
**Hiroshima**	97.0±1.1 b	147.9±1.9 bc	198.8±6.3 cd	9,240±293.6 ab	2.2±0.0 f
**Saga 1**	104.3±1.4 a	155.6±1.7 a	222.1±6.4 abcd	8,647±228.6 abc	2.6±0.0 e
**Saga 2**	98.0±1.3 b	146.8±2.2 c	267.0±9.6 ab	8,163±253.0 abc	3.2±0.1 b
**Saga 3**	96.9±1.2 b	150.1±2.0 b	247.6±7.0 abc	9,558±274.8 a	2.6±0.0 de
**Across populations**	93.1±0.5	146.8±0.7	237.1±3.1	8,498±96.5	2.8±0.0
**Fold difference** ^**c**^	1.5	1.3	1.5	1.3	1.6

For each characteristic, significant differences at 0.05 level are designated by different letters.

^a^ Flowering and maturity were expressed in days after planting (DAP).

^b^ Seed size was expressed as 100 seed weight.

^c^ Fold difference between minimum and maximum values observed for different populations.

Significant differences were observed for wild soybean yield that ranged from 179.8 to 275.3 g across populations (Table 3). Variance component analysis indicated that variation in yield was due to a lesser extent by the genotype (8.8%) and more by environmental effects (15.4%) and G x E interaction (15.3%) (Fig 1).

The number of seeds varied across populations ranging from 7,162 to 9,558 seeds per plant (Table 3). This variation was predominantly due to environmental effects (17.2%) and G x E interaction (13.7%) (Fig 1).

Seed size varied significantly ranging from 2.2 to 3.2 g across populations (Table 3). Variation was mostly due to genetic differences among populations (37.9%), whereas the environmental effect (12.3%) and G x E interaction (11.8%) had comparatively lesser impact on this characteristic (Fig 1).

### Comparison among plant densities

The Aomori 1 population was evaluated using different planting densities under ruderal and natural growing conditions (Table 4). When comparing planting densities, similar trends were noted for observed characteristics for both growing conditions. Plant emergence rate, end of season plant survival, and seed size were not impacted by different planting densities (Table 4). When expressed per plot, seed number and yield showed no significant differences among planting densities. However, when expressed per plant, both seed number and yield had higher values in lower density plots, even though these differences were not significant in all cases (Table 4).

**Table 4:** Wild soybean values (mean±SE) across planting densities under ruderal and natural growing conditions.

Density (seeds/m^2^)	Plant emergence (%)	End season survival (%)	Yield (g)		Seed number		Seed size (g) ^a^
			Per plot	Per plant	Per plot	Per plant	
**Ruderal growing conditions**							
**112**	67.9±11.7 b	82.8±14.7 a	49.8±15.9 a	15.1±6.3 a	1107.6±458.8 a	347.4±171.4 a	2.9±0.3 a
**208**	81.7±7.1 a	77.6±9.1 a	70.7±24.0 a	10.2±4.2 ab	1695.0±687.3 a	240.7±109.6 ab	2.9±0.1 a
**416**	79.8±6.8 ab	75.2±9.4 a	67.7±19.6 a	5.1±1.6 b	1454.8±513.7 a	109.9±40.7 bc	3.0±0.2 a
**832**	76.4±8.9 ab	83.4±6.3 a	72.9±20.9 a	3.5±1.4 b	1765.8±630.2 a	83.1±37.3 c	2.8±0.1 a
**Fold difference** ^**b**^	1.2	1.1	1.7	4.3	1.6	4.2	1.1
**Natural growing conditions**							
**112**	57.1±9.4 a	36.6±15.6 a	10.3±5.4 a	2.0±0.9 a	369.8±195.9 a	72.7±32.0 a	3.0±0.2 a
**208**	68.3±7.9 a	29.7±14.1 a	6.4±4.5 a	0.9±0.7 a	196.8±129.0 a	26.5±18.8 ab	3.0±0.2 a
**416**	63.5±8.7 a	22.9±12.0 a	24.5±23.3 a	1.4±1.3 a	569.3±522.9 a	33.3±28.5 ab	3.2±0.6 a
**832**	63.2±8.3 a	24.9±12.4 a	4.9±4.3 a	0.2±0.1 a	161.7±135.6 a	5.2±4.2 b	2.8±0.4 a
**Fold difference** ^**b**^	1.2	1.6	5.0	12.9	4.6	14.0	1.1
**Across growing conditions**							
**112**	62.5±7.4 a	59.7±11.9 a	35.4±11.7 a	10.3±4.4 a	910.9±346.5 a	274.1±128.5 a	3.0±0.2 a
**208**	75.0±5.4 a	53.6±10.2 a	49.3±18.2 a	7.1±3.0 a	1320.4±537.3 a	187.1±84.9 b	2.9±0.1 a
**416**	71.6±5.7 a	49.1±10.0 a	53.3±15.8 a	3.9±1.2 a	1233.4±410.6 a	90.7±32.0 bc	3.0±0.2 a
**832**	69.8±6.1 a	54.1±10.1 a	54.4±17.7 a	2.6±1.1 a	1444.9±528.8 a	67.5±30.8 c	2.8±0.1 a
**Fold difference** ^**b**^	1.2	1.2	1.5	4.0	1.6	4.1	1.1

For each growing condition and characteristic, significant differences at 0.05 level among plant densities are designated by different letters.

^a^ Seed size was expressed as 100 seed weight.

^b^ Fold difference between minimum and maximum values observed for different planting densities.

When comparing planting densities, similar trends were observed for leaf coverage of wild soybean under both ruderal and natural growing conditions (Table 5). Across growing conditions, plots with lower planting density had less wild soybean leaf coverage early in the season, whereas by October leaf coverage generally did not differ among planting densities. However, when expressed per plant, leaf coverage at the end of the season was significantly reduced as planting density increased (Table 5).

**Table 5:** Leaf coverage of wild soybean (mean±SE) during the season across planting densities under ruderal and natural growing conditions.

Density (seeds/m^2^)	Leaf coverage (%)					
	June-July	July	August	September	October	Per plant ^a^
**Ruderal growing conditions** (5.8%) ^b^						
**112**	0.7±0.2 b	1.3±0.3 b	7.2±2.0 b	8.4±1.9 b	6.3±1.1 a	1.3±0.3 a
**208**	0.7±0.2 b	1.3±0.3 b	12.2±3.7 a	11.3±2.9 a	5.6±1.3 a	0.8±0.2 b
**416**	1.0±0.3 a	1.7±0.4 a	10.3±2.6 ab	11.5±2.6 a	5.6±1.3 a	0.4±0.1 c
**832**	1.1±0.3 a	1.6±0.3 ab	11.5±3.4 a	11.0±2.6 ab	5.5±1.1 a	0.2±0.0 c
**Fold difference** ^**c**^	1.6	1.3	1.7	1.4	1.1	6.5
**Natural growing conditions** (2.3%) ^b^						
**112**	0.4±0.2 b	1.4±0.3 a	2.2±1.0 a	3.2±1.4 a	4.3±1.8 a	0.3±0.0 a
**208**	0.7±0.3 a	1.4±0.2 a	2.8±1.0 a	2.8±1.0 a	3.4±1.2 a	0.2±0.1 ab
**416**	0.8±0.3 a	1.7±0.3 a	3.0±1.2 a	3.6±1.7 a	4.4±1.6 a	0.2±0.1 ab
**832**	0.9±0.3 a	1.8±0.3 a	2.6±0.9 a	3.1±1.4 a	4.4±1.9 a	0.05±0.0 b
**Fold difference** ^**c**^	2.3	1.3	1.4	1.3	1.3	6.1
**Across growing conditions **						
**112**	0.5±01 c	1.4±0.2 b	4.7±1.2 a	5.8±1.3 b	5.3±1.1 a	1.0±0.3 a
**208**	0.7±0.2 bc	1.4±0.2 b	7.5±2.1 a	7.0±1.7 ab	4.5±0.9 a	0.6±0.1 b
**416**	0.9±0.2 ab	1.7±0.3 a	6.6±1.6 a	7.5±1.7 a	5.0±1.0 a	0.3±0.1 bc
**832**	1.0±0.2 a	1.7±0.2 a	7.0±1.9 a	7.1±1.7 ab	5.0±1.1 a	0.1±0.0 c
**Fold difference** ^**c**^	1.8	1.3	1.3	1.2	1.2	7.2

For each growing condition and leaf coverage observation, significant differences at 0.05 level among plant densities are designated by different letters.

^a^ Wild soybean leaf coverage per plant at the end of the season.

^b^ Mean leaf coverage values of wild soybean during the season.

^c^ Fold difference between minimum and maximum values observed for different planting densities.

Across growing conditions and within each observation, leaf coverage for the other plot vegetation was very comparable among plots, *i*.*e*., it was not impacted by different planting densities of wild soybean (Table 6). For both ruderal and natural growing conditions, average leaf coverage values for non-wild soybean vegetation (57.9% and 65.0%, respectively) were significantly higher than those for wild soybean (5.8% and 2.3%, respectively) (Tables 5 and 6). This trend was true for individual comparisons regardless of growing conditions, plant densities, or observation times.

**Table 6:** Leaf coverage of other plot vegetation (mean±SE) during the season across wild soybean planting densities under ruderal and natural growing conditions.

Density (seeds/m^2^)	Leaf coverage (%)				
	June-July	July	August	September	October
**Ruderal growing conditions** (57.9%) ^a^					
**112**	19.7±9.1 a	26.6±7.2 a	57.5±7.3 a	83.6±2.0 a	90.3±1.1 a
**208**	17.0±6.4 a	26.2±7.4 a	65.3±4.4 a	84.8±1.6 a	87.8±2.2 a
**416**	20.4±8.2 a	27.9±6.5 a	57.3±6.9 a	83.5±2.6 a	87.5±0.9 a
**832**	22.3±9.6 a	28.1±7.5 a	59.4±6.6 a	82.8±2.2 a	86.9±2.2 a
**Fold difference** ^**b**^	1.3	1.1	1.1	1.0	1.0
**Natural growing conditions** (65.0%) ^a^					
**112**	52.5±6.9 a	54.1±7.3 a	62.3±6.1 a	82.9±3.8 a	79.6±3.3 a
**208**	56.5±6.8 a	60.4±5.9 a	64.6±5.3 a	81.9±3.9 a	75.0±4.2 a
**416**	51.6±4.9 a	55.5±7.3 a	62.6±5.0 a	75.4±5.2 b	78.1±4.9 a
**832**	51.3±7.5 a	60.8±5.8 a	71.9±5.1 a	82.8±3.5 a	78.8±2.8 a
**Fold difference** ^**b**^	1.1	1.1	1.2	1.1	1.1
**Across growing conditions**					
**112**	39.4±6.5 a	43.1±6.0 a	60.4±4.6 a	83.3±1.9 a	84.6±2.3 a
**208**	40.7±6.5 a	46.7±5.9 a	64.9±3.6 a	83.5±1.8 a	81.4±2.8 a
**416**	39.1±5.5 a	44.5±5.8 a	60.5±4.0 a	80.2±2.7 a	82.8±2.7 a
**832**	39.7±6.6 a	47.7±5.8 a	66.9±4.2 a	82.8±1.9 a	82.8±2.0 a
**Fold difference** ^**b**^	1.0	1.1	1.1	1.0	1.0

For each growing condition and leaf coverage observation, significant differences at 0.05 level among plant densities are designated by different letters.

^a^ Mean leaf coverage values of other plant vegetation during the season.

^b^ Fold difference between minimum and maximum values observed for different planting densities.

### Comparison among growing conditions

Seed production of Aomori 1 was evaluated under different growing conditions allowing for comparisons among favorable, ruderal, and natural environments. Wild soybean plants yielded significantly more under favorable conditions (271.8 g) than under ruderal (5.3 g) or natural growing conditions (1.1 g), representing a 51-fold and a 247-fold yield reduction, respectively (Table 7). Similarly, significantly more seeds were produced by plants under favorable conditions (7,625.6) than under ruderal (124.2) and natural conditions (31.8), representing a 61-fold and a 240-fold reduction, respectively. No significant differences were observed for seed size among the three growing conditions (Table 7).

**Table 7:** Comparison (mean±SE) of seed production characteristics (yield per plant, seed number per plant, and seed size) of Aomori 1 population grown under favorable, ruderal, and natural conditions.

Growing conditions	Yield (g)	Seed number	Seed size (g) ^a^
**Favorable**	271.8±15.7 a	7,625.6±404.2 a	3.6±0.1 a
**Ruderal**	5.3±1.7 b	124.2±48.0 b	3.0±0.1 a
**Natural**	1.1±0.6 b	31.8±16.3 b	3.1±0.3 a
**Fold difference between favorable and ruderal conditions**	51	61	1.2
**Fold difference between favorable and natural conditions**	247	240	1.2

For each characteristic, significant differences at 0.05 level are designated by different letters.

^a^ Seed size was expressed as 100 seed weight.

## Discussion

### Comparison across populations under favorable conditions

Diversity among the 11 populations used in this study was assumed as the populations originated from different regions in Japan including northern (Aomori), central (Ibaraki), and southern (Hiroshima and Saga) prefectures. The wide range in flowering (70.0–104.3 DAP) and maturity (120.1–155.6 DAP), each spanning over a month, confirmed genetic diversity among the wild soybean entries. As a comparison, Dong *et al*. [20] reported that 48.2% of evaluated wild soybean accessions from a germplasm collection from China matured within a similar range (120–140 DAP).

Results indicated that, besides genetic factors, environmental conditions were important sources of variation for both flowering and maturity. This is not surprising considering that wild soybean shows strong photoperiod sensitivity [21] resulting in environmental factors like daylength, planting time, or seasonal temperatures influencing the time to flowering and maturity for the species. In the current study, all populations showed substantially longer time to flowering and maturity at sites planted mid-May compared to those planted mid-June (S1 Table). Tsubokura *et al*. [22] observed a similar trend for a wild soybean population they evaluated. This is consistent with results observed for cultivated soybean where growing conditions (*e*.*g*., daylength, time of planting, or seasonal temperature) play a significant role in time to flowering and maturity [22–24].

Whereas the impact of different environments (sites and years) was substantial, the variation among replications within a given population at a given site was very limited for flowering and maturity. Consequently, residual variance accounted for only a small portion of the total variation for the two characteristics. Similarly, Cregan and Hartwig [24] have shown low estimates of error term for flowering time in soybeans.

The evaluation of seed production under favorable growing condition was intended to measure potential of yield and yield components per plant at minimum levels of stress. The results indicated a high yield potential of wild soybean grown under favorable environmental conditions. Across populations the average yield per plant was 237.1 g and was mostly due to a high number of seeds per plant (average of 8,478 seeds per plant), rather than large seed size (average of 2.8 g). The seed production potential of individual plants showed yield of up to 763.5 g (for Aomori 1, first replication at 2013IB), number of seeds of up to 19,909 (for Ibaraki 4, second replication at 2012IB1), and seed size of up to 5.3 g (for Aomori 1, second replication at 2012IB2) (S2 Table).

Significant differences across populations were observed for all three seed production characteristics. Variance component analysis indicated that environmental factors and G x E interaction influenced variation of yield and seed number to a greater extent than the genotype. Conversely, variation in seed size was mostly due to genotype rather than to either environmental effects or G x E interaction. It should be noted that for all three characteristics, the genetic portion of plant-to-plant variation within populations could not be estimated as the study was designed to assess genetic differences among populations. The residual effect had high values, which can be observed for highly variable characteristics such as yield [18].

### Comparison among plant densities

Comparison among wild soybean plots planted at different densities indicated the importance of intra-specific competition. Though not statistically significant in all cases, yield per plant tended to increase with reduced planting density. Specifically, a 50% reduction in plant population resulted in an average of 59% yield increase per plant. Similarly, Yoda *et al*. [12] estimated that 50% reduction in plant density resulted in 41% increase in plant yield.

Of the two yield components, it was seed number, not the seed size that impacted the observed yield trend. Plots with fewer wild soybean plants produced significantly more seeds per plant. In contrast, seed size did not change regardless of planting density.

The plant survival values were comparable across different planting densities. In contrast, others have shown a reduction of soybean plant survival with increased plant population [12,13]. This discrepancy might be because plants in those studies were exposed to intra-specific competition only, whereas in the current study wild soybean was exposed not only to intra-specific competition, but also to the inter-specific competition with similar plant competitive pressure across planting densities.

Leaf coverage per wild soybean plant was reduced significantly as the planting density increased. This was observed for both ruderal and natural growing conditions. The same trend was reported for cultivated soybean where plots with higher population densities experienced increased leaf loss at the end of the season [15].

### Comparison among growing conditions

Comparison among the three growing conditions indicated large differences in yield. Wild soybean plants produced on average 271.8 g of seeds under favorable conditions, which was 51 and 247 times more than what was produced under ruderal and natural growing conditions, respectively. Such a substantial decrease in yield can be attributed to sub-optimal conditions including inter-specific plant competition. Under favorable growing conditions without plant competitive pressure from other species, 93.1% of wild soybean plants survived (the plant loss occurred mostly at the site where rabbit damage was observed early in the season). In contrast, survival of wild soybean plants under ruderal and natural growing conditions was reduced to as low as 75.2% and 22.9%, respectively.

Leaf coverage of other vegetation also differed across the three growing conditions, ranging from no leaf coverage under favorable conditions to over 80% leaf coverage in both ruderal and natural fields later in the season (September and October). This inter-specific competition negatively impacted seed production of wild soybean. The yield per plant was only 5.3 g under ruderal conditions and 1.1 g under natural conditions. The data illustrate the extent of impact that environmental factors have on seed production. Wild soybeans have enormous potential to yield, but this potential has not been realized in nature due to sub-optimal environmental conditions.

Similar trends were observed for number of seeds per plant. The wild soybean plants produced on average 7,625.6 seeds per plant under favorable conditions, which was 61 and 240 times more than what was produced under ruderal and natural growing conditions, respectively (Table 7). The seed number per plant decreased significantly to 124.2 under ruderal conditions and 31.8 under natural conditions indicating again the extent of the impact of environmental stressors.

In contrast to yield and seed number, seed size was not significantly impacted by environmental conditions as sub-optimal growing conditions resulted in relatively minor and statistically not significant reduction in seed size compared to plants grown under favorable conditions.

## Conclusions

In conclusion, the results indicate that high variability associated with yield in wild soybean is influenced mostly by environmental factors and G x E interaction. Both intra- and inter-specific plant competition impacted seed production of wild soybean. Reduction of yield under suboptimal condition is expected, but this study showed the extent of that reduction in wild soybean, as well as the trends associated with the key yield components. The high yield potential was substantially reduced in sub-optimal, natural environments (up to 247-fold) mostly due to decrease in seed number (up to 240-fold).

The current study contributes to a better understanding of yield potential and variability associated with seed production in wild soybean and should be considered when assessing the potential impact of soybean biotechnology traits that increase yield or protect against yield loss. If such traits were to be inadvertently transferred from imported soybean into wild soybean, this research indicates that the effects would likely have little overall impact on wild soybean seed production. This is due to substantial seed production potential and observed variability of wild soybean when compared to the modest levels of yield increase potentially provided by a transferred biotechnology trait.

## Supporting information

S1 Table Flowering and maturity averaged per location and across wild soybean populations grown under favorable growing conditions. (DOCX)

S2 Table Maximum values observed for individual plants for yield, seed number, and seed size of wild soybean grown under favorable growing conditions. (DOCX)
