# Supplementary material for: Seed production of wild soybean (Glycine soja Sieb. et Zucc.) under favorable, ruderal, and natural growing conditions
Source: PLoS One. 2022 Sep 29;17(9):e0274668. doi: 10.1371/journal.pone.0274668 (PMC9522317; doi:10.1371/journal.pone.0274668)
Supplement: S1 Table — (DOCX) [file pone.0274668.s001.docx]

S 1 Table. Flowering and maturity averaged per location and across wild soybean populations grown under favorable growing conditions.

| **Locations** | **Planting Date** | **Flowering (DAP) ^a^** | **Maturity (DAP) ^a^** |
| --- | --- | --- | --- |
| **2012IB1** | 14 May | 103.3 | 163.7 |
| **2012IB2** | 16 May | 105.0 | 163.1 |
| **2013IB** | 4 June | 81.7 | 140.8 |
| **2013FU** | 18 June | 84.5 | 123.3 |

^a^ Flowering and maturity were expressed in days after planting (DAP).
