# Supplementary material for: Seed production of wild soybean (Glycine soja Sieb. et Zucc.) under favorable, ruderal, and natural growing conditions
Source: PLoS One. 2022 Sep 29;17(9):e0274668. doi: 10.1371/journal.pone.0274668 (PMC9522317; doi:10.1371/journal.pone.0274668)
Supplement: S2 Table — (DOCX) [file pone.0274668.s002.docx]

S 2 Table. Maximum values observed for individual plants for yield, seed number, and seed size of wild soybean grown under favorable growing conditions.

| **Population** | **Yield (g)** | **Seed number** | **Seed size (g) ^a^** |
| --- | --- | --- | --- |
| **Aomori 1** | 763.5 | 15,600 | 5.3 |
| **Aomori 2** | 341.3 | 14,138 | 3.6 |
| **Ibaraki 1** | 497.1 | 15,822 | 4.1 |
| **Ibaraki 2** | 483.4 | 15,013 | 4.1 |
| **Ibaraki 3** | 372.1 | 12,915 | 3.5 |
| **Ibaraki 4** | 534.7 | 19,909 | 3.6 |
| **Ibaraki 5** | 363.3 | 14,368 | 3.0 |
| **Hiroshima** | 315.9 | 15,218 | 2.7 |
| **Saga 1** | 433.2 | 13,912 | 3.8 |
| **Saga 2** | 462.5 | 13,705 | 3.8 |
| **Saga 3** | 479.6 | 16,972 | 3.6 |

^a^ Seed size was expressed as 100 seed weight.
